# Unusual feature of neonatal hypernatremic dehydration due to microvillus inclusion disease: a case report

**DOI:** 10.11604/pamj.2018.30.109.12330

**Published:** 2018-06-11

**Authors:** Fatma Khalsi, Mohamed Riadh Boukhris, Ines Brini, Jean Pierre Hugot, Patsy Dominique Berrebi, Khedija Boussetta

**Affiliations:** 1 Pediatric Department B, Children’s Hospital of Tunis, Tunisia; 2 Gastroenterology Department, Robert Debré Hospital, Paris, France; 3 Cytology Department, Robert Debré Hospital, Paris, France

**Keywords:** Neonate, dehydration, micovillus inclusion disease

## Abstract

All over the causes of intractable diarrhea of infancy, microvillous inclusion disease is a rare congenital defect of intestinal brush border of unknown aetiology. An autosomal recessive inheritance is suggested by cases occurring in siblings and high incidence of consanguinity. The prognosis of the disease is extremely poor, as life can be sustained only by total parenteral nutrition. The authors report a preterm male newborn of 35 weeks gestation presenting severe hypernatremic dehydration on day 4 after birth caused by a secretory profuse diarrhea and discuss the tools allowing the light microscopic and genetic diagnosis. The final diagnosis of microvillus intestinal disease (MVID) was made on the third month after extensive investigations using electron microscopic examination of intestinal biopsy and genetic confirmation, finding a mutation at the homozygous status of MYO5B gene. The infant died on the fourth month in spite of optimal electrolytic support and parenteral prolonged nutrition. Although MVID is extremely rare, it remains a possible cause of intractable secretory diarrhea leading to severe hypernatremic dehydration and metabolic acidosis in neonates.

## Introduction

The dehydration is a rare symptom in neonatal period with a various etiology.Microvillus atrophy disease (MVID; OMIM251850) is an extremely rare and severe enteropathy causing protracted diarrhea starting at the first few days of life. We report a case of neonatal dehydration secondary to profuse diarrhea due to MVID. The management of this condition and the positive diagnosis were difficult. Through this case, we will try to explain the clinical aspects of MVID and highlight the importance of early diagnosis.

## Patient and observation

We report a case of a male near-term neonate with gestational age of 35 weeks. He was the third born child to a 27-year-old mother with no significant past history. He was derived from a third degree consanguineous marriage. He was born by means of normal vaginal delivery with a normal extra-uterine adaptation. Meconium-stained amniotic fluid was noted. His birth weight was 3030g (75^th^ Percentile). The baby had normal physical examination at birth aside transitory respiratory distress. He had been hospitalized for four days in a private clinic, for the suspicion of materno-foetal infection, where he had antibiotics for 48 hours. He had developed no symptoms during his hospitalization. The output's weight was 2400g which signify the loss of 630g. Back home, he received exclusive maternal breastfeeding.On his fifth day of life, he presented multiple yellowish-watery stools (ab 12 times) and refuse feeding with apyrexia context. He had clinical signs of severe dehydration without fever or vomiting. He lost 930g relative to the birth?s weight. The abdomen appeared neither tender nor distended, and the rest of his physical examination was normal. The laboratory findings revealed hypernatremia of 152mEq/L, hyperkalemia of 7,8mEq/L and severe metabolic acidosis: blood PH: 7,1, HCO3:4mEq/L. The osmotic gap was 45. There was no leukocytosis, and the serum level of C-reactive protein was normal.The infant was treated with intravenous fluids and electrolytes as well as antibiotics after obtaining blood and stool cultures, assuming an infectious etiology. After 26 hours of rehydration therapy, the dehydration resolved, however, a functional acute renal failure complicated by acidosis and hyperkalemia appeared. The chest radiograph showed “bat wings” pattern and the cardiothoracic index was 0, 45.The renal ultrasound showed discretely hyperechoic kidneys cortex and the absence of renal malformations. On the 11^th^ day of life, he was transferred to the pediatric intensive care unit. He needed mechanical ventilation for three days and exclusive parenteral feeding by central catheter. The evolution was burdened by cholestasis due to the prolonged parenteral feeding and nosocomial infection by staphylococcus epidermidis. Since his admission, he had developed a slimy diarrhea with 3 to 4 stools per day. He presented a profuse diarrhea during each attempt to introduce enteral feeding by hydrolyzed milk formula. He was discharged after 32 days with output's weight 3250g. The oesophago-gastro-duodenoscopy and duodenal biopsy showed partial villous atrophy associated to the brush border abnormalities. The periodic acid Schiff (PAS) stain revealed abnormalities in the brush border characterized by loss of the linear part of enterocytes with the presence of intra-cytoplasmic PAS + band in the apical pole of enterocytes uneven thickness and sometimes with double contours pattern. In the submucosa, there were numerous Brunner glands. We find the existence of numerous hemorrhagic suffusions. The CD10 immunostain was positive. Thus, a diagnosis hypothesis of MVID was raised. The electron microscopic examination confirmed the diagnosis. Mutation analysis of peripheral blood samples of the neonate revealed a mutation at the homozygous status of MYO5B gene. The baby was died, after 97 days of life, of septic shock and multiple organ failure.

## Discussion

Neonatal dehydration is an uncommon problem with a variety of etiologies that presents particularly difficult diagnostic and therapeutic challenges. MVID may be one of these etiologies. It is one of congenital enterocyte disorders that causes severe and intractable secretory diarrhea leading to severe dehydration [1]. The MIVD is extremely rare. The incidence in the Japanese population is about of 1 per 3,000,000 live births [2]. A female preponderance of 2:1 has been reported (three). Two forms are described: early onset MVID diarrhea beginning in the first 72 hours of life, and late onset with first symptoms appears at 6-8 weeks after birth [3]. In our case, the diarrhea began early in life at the fifth day. MVID was first described by Davidson et al in 1978 as familial enteropathy presenting with protracted diarrhea from birth, failure to thrive and hypoplasic villus atrophy [4,5] The secretory diarrhea is always the common symptom with a remarkably raised level of faecal sodium. However, enteral feeding can cause osmotic diarrhea [1]. Rapid and severe dehydration is inevitable unless intravenous rehydration is undertaken. In our case, the newborn necessitated aggressive resuscitation to curb the symptoms. The diagnosis rested on the ultrastructural findings on the electron microscopy of a partial to total atrophy of microvilli on mature enterocytes with apical accumulation of numerous secretory granules in immature enterocytes; and the highly characteristic inclusion bodies containing rudimentary or fully differentiated microvilli in mature enterocytes. Light microscopy shows accumulation of PAS-positive granules at the apical pole of immature enterocytes, together with atrophic band indicating microvillus atrophy and, in parallel, an intracellular PAS or CD10 positive line [6]. Lightened electron microscopic findings in our patient's jejunumspecimens are consistent with stated above. This disorder has an autosomal recessive inheritance. Regarding the high consanguinity rate in our region, the disease is probably under reported. MVID is caused by mutations in the MYO5B gene (OMIM 606540) on chromosome 18q21 that defect intracellular traffic and disrupt epithelial cell polarity [7]. MYO5B gene encodes myosin Vb that regulates membrane trafficking along the recycling pathway in polarized epithelial cells [8]. The MVID patients are at risk of developing a PFIC-like liver disease [9]. The cholestasis results from the impairment of the MYO5B/RAB11A apical recycling endosome pathway in hepatocytes, the altered targeting of BSEP to the canalicular membrane and the increased ileal bile acid absorption [9]. Our patient had developed a cholestasis on the first month of life.A new study describes the appearance of necrotizing enterocolitis in an infant with MVID [10]. There is no curative therapy. The only proved treatment, that can give hope, is small bowel transplantation alone or in combination with liver that improves the outcome and quality of life [10]. In our case, the infant hadn't a specific treatment of MVID because of lack of transplantation opportunities in our country. The antenatal diagnosis can be suspected in front of hyperechoic bowel loops in the systemic antenatal ultrasound [4] or bowel dilation with polyhydramnios [4,10]. Molecular analysis of the MYO5B gene is helpful in genetic counseling and prenatal diagnosis of recurrent microvillus inclusion disease in subsequent pregnancies [4,5]. The prognosis of MVID is poor. The majority of cases with early onset disease die between 3 and 9 months of life due to dehydration, malnutrition and sepsis [1]. Our newborn had a very early beginning of the manifestations and he died in the sixth month of life due to sepsis.

## Conclusion

The microvillus intestinal disease is a life threatening condition leading to a poor outcome. With the recent advances of ultrasound techniques, antenatal diagnosis becomes possible which can improve the prognosis. Combined bowel-liver or bowel transplantation is regarded as the only potentially life-saving therapy.

## Competing interests

The authors declare no competing interests.
